# Biomechanical effects of loading methods on the patellofemoral joint during stair climbing: based on statistical parametric mapping analysis

**DOI:** 10.3389/fbioe.2025.1617823

**Published:** 2025-06-09

**Authors:** Hongwen Zhang, Xingchen Zhang, Jing Ma, Na Sun, Litai Zhang, Yuan Gao

**Affiliations:** ^1^ Integrated Traditional Chinese and Western Medicine Rehabilitation Department, PLA Joint Logistics Support Force Beidaihe Rehabilitation and Convalescence Center, Qinhuangdao, Hebei, China; ^2^ School of Physical Education, Yanshan University, Qinhuangdao, Hebei, China; ^3^ Key Lab of Intelligent Rehabilitation and Neuroregulation in Hebei Province, Yanshan University, Qinhuangdao, Hebei, China

**Keywords:** statistical parametric mapping analysis, patellofemoral joint, Stairs, load carrying method, Stability

## Abstract

**Introduction:**

Stair negotiation with external loads imposes substantial demands on the structural and functional integrity of the patellofemoral joint. Current research predominantly focuses on singular loading modalities or level walking conditions, often employing discrete time-point comparisons. This study innovatively employs Statistical Parametric Mapping (SPM) to systematically analyze patellofemoral biomechanical characteristics during stair negotiation with different load-carrying strategies.

**Methods:**

Twenty healthy males performed stair negotiation tasks under shoulder-load carriage (SLC) and hand-carry carriage (HCC) conditions (15 kg). Kinematic (200 Hz), kinetic (2000 Hz), and electromyographic (2000 Hz) data were synchronized to compute patellofemoral joint stress(PFJS), center of pressure (COP) trajectories, and muscle co-activation indices across stair phases.

**Results:**

HCC generated significantly greater patellofemoral joint stress during most stair phases compared to SLC (*P* < 0.05), while SLC exhibited transient stress elevation only during initial double-support phase.

**Discussion:**

HCC particularly increased joint stress during single-support and second double-support phases, with concomitant increases in COP displacement distances and reduced lower-limb co-cativation indices (CCI) collectively compromising joint stability. Despite transient stress spikes during initial double-support, SLC maintained kinetic chain equilibrium through shorter external moment arms. These findings recommend prioritizing proximal symmetric loading modes complemented by targeted vastus medialis training to enhance patellar stability, thereby reducing both patellofemoral joint stress concentrations and low back pain risks.

## 1 Introduction

With the evolution of occupational activities and lifestyles in modern society, load-bearing stair ascent and descent have become routine tasks for specific populations, including construction workers, mountaineering enthusiasts, and individuals in their daily lives. Among load carriage methods, shoulder-load carriage (SLC) and hand-carry carriage (HCC) are the most frequently encountered in daily life (Geng, et al., 2023). This activity imposes significant repetitive high-impact loading on the knee joint, particularly the patellofemoral joint, thereby elevating functional and structural demands on the patellofemoral articulation (Nadeau S. et al., 2003). Relevant studies have demonstrated that patellofemoral joint stress (PFJS) during stair negotiation is 2–4 times significantly higher than during level-ground walking, which is closely associated with increased knee joint moments and elevated patellofemoral joint contact forces during stair negotiation (Novak and Brouwer, 2011). This mechanism has been conclusively established in the pathogenesis of patellofemoral pain syndrome (Nunes et al., 2018). Patellofemoral pain syndrome affects approximately 20% of adults and 30% of adolescents (Garcia-Hermoso et al., 2023), females exhibit a higher prevalence of patellofemoral pain syndrome compared to males (It may be due to the larger Q-angle and shallower femoral trochlea in females.) (Lankhorst et al., 2012), with significantly higher prevalence rates observed in individuals engaged in prolonged stair-loading tasks compared to the general population (Andersen et al., 2007).

Studies have demonstrated that external loading significantly increases knee joint moments (Simpson et al., 2011), resulting in elevated PFJS. But existing research has predominantly focused on single-load configurations or level walking conditions (Kinoshita, 1985; Attwells et al., 2006). However, although distinct loading modalities during stair negotiation may alter center of mass (COM) positioning and muscle activation patterns (Kim and Song, 2012) and exert differential impacts on PFJS, the underlying mechanisms remain incompletely elucidated. To fill this translational gap, we contrast shoulder-load carriage and hand-carry carriage modalities—the two most prevalent yet biomechanically divergent modalities—to identify which strategy minimizes patellofemoral loading during stair negotiation, a critical input for evidence-based decision-making in occupational health and rehabilitation. In addition, traditional biomechanical analyses predominantly utilize discrete time points (e.g., characteristic peaks) for comparative studies, which fail to comprehensively reveal dynamic stress variation patterns throughout movement cycles (Wang et al., 2020). Single peak PFJS values may inadequately assess patellofemoral pain risk due to two key limitations: (1) ignorance of sustained stress accumulation and cumulative load magnitude over time and (2) oversimplification of dynamic loading patterns. In contrast, Statistical Parametric Mapping (SPM) overcomes data extraction biases through continuous time-series hypothesis testing; statistical results are directly displayed in the original sampling space, making the spatiotemporal biomechanical context immediately clear. Furthermore, the analysis does not require preset assumptions regarding the spatiotemporal foci of the analyzed signals, this methodology has been widely implemented in diverse biomechanical research domains (Pataky et al., 2013; Richter et al., 2014).

Based on this,the current study applies Statistical Parametric Mapping (SPM) to systematically compare the dynamic characteristics of patellofemoral joint stress, center of pressure (COP), and muscle co-cativation indices (CCI) between HCC and SLC modalities during stair negotiation. These findings provide evidence-based guidelines for optimizing load carriage strategies, developing injury prevention protocols, and mitigating occupational knee injury risks.

## 2 Methods

### 2.1 Participants

A priori power analysis was conducted in G*Power 3.1 with a medium effect size (Cohen’s d = 0.5), an alpha level of 0.05 (two-tailed), and a power of 0.8, yielding a minimum sample size of 20 participants. Thus, this study recruited 20 male university students (height: 182.02 ± 4.35 cm, weight: 70.69 ± 7.85 kg, age: 18.68 ± 0.86 years) meeting the following inclusion criteria: 1) No history of chronic diseases and normal neuromusculoskeletal function; 2) No strenuous exercise within 24 h prior to testing and absence of muscle fatigue symptoms; 3) No significant lower extremity injuries within the preceding 6 months. All participants provided written informed consent after being fully informed of the study objectives and experimental protocols. Voluntary participation was ensured through standardized ethical approval procedures.

### 2.2 Experimental procedures

**FIGURE 1:**
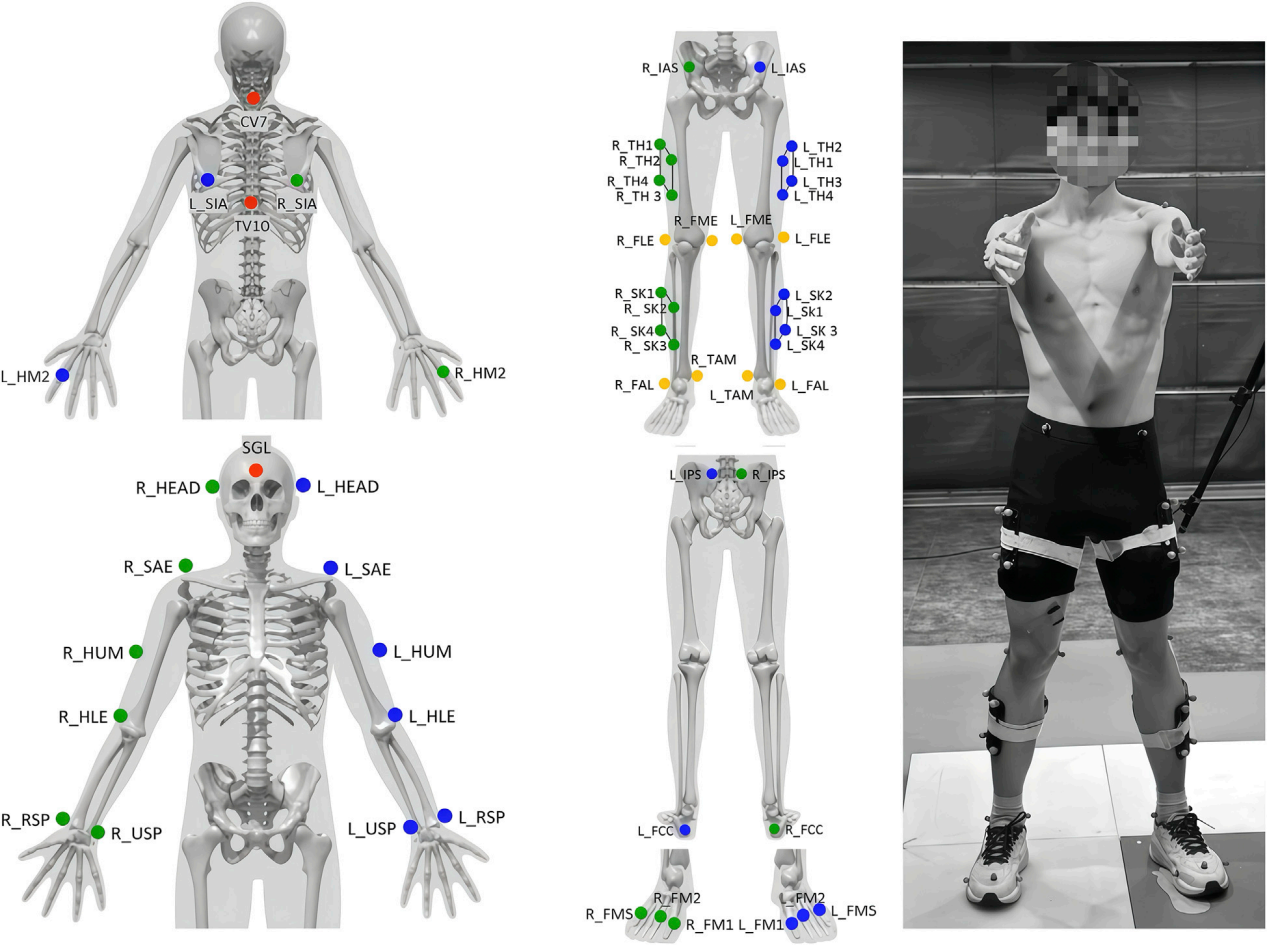
Placement positions of reflective markers.

The experimental set up incorporated four Kistler force platforms (9260AA6; 2,000 Hz), eight Qualisys infrared high-speed cameras (Arqus; 200 Hz), and a Delsys surface electromyography system. All devices were connected to a central analog-to-digital converter and synchronized via a 5 V TTL to ensure temporal alignment during data collection. Prior to electrode placement, skin preparation included shaving hair and cleansing with 75% ethanol to reduce impedance, followed by air-drying. Operators then positioned electromyography (EMG) electrodes and reflective markers at designated anatomical landmarks following SENIAM guidelines. The monitored muscles included vastus lateralis (VL), rectus femoris (RF), vastus medialis (VM), tibialis anterior (TA), biceps femoris (BF), lateral gastrocnemius (LG), and medial gastrocnemius (MG). EMG electrode positions are specified in Table 1. Prior to testing, three-dimensional spatial calibration of the measurement volume was performed. Following spatial calibration, a total of 55 reflective markers s (39 anatomical markers +16 tracking clusters) were affixed according to the Qualisys Lower Limb Marker Protocol (Qualisys, 2023), with detailed placements illustrated in Figure 1. Following these preparations, experimental trials were conducted.

**TABLE 1:** Muscle identification and electrode placement.

Name	Electrode positions
vastus lateralis (VL)	2/3 distally along the line from the anterior superior iliac spine to the lateral patellar border
rectus femoris (RF)	Midpoint of the line between the ASIS and the superior patellar pole
vastus medialis (VM)	4/5 distally along the line from the ASIS to the medial joint space of the knee
tibialis anterior (TA)	At 25%–33% of the leg length (from knee joint line to lateral malleolus), lateral to the tibial crest
biceps femoris (BF)	Midpoint between the ischial tuberosity and the lateral tibial condyle
lateral gastrocnemius (LG)	Posterior to the medial femoral condyle, 1/3 along the line from the fibular head to the calcaneus
medial gastrocnemius (MG)	Over the maximal muscle bulge between the medial femoral epicondyle and the calcaneal tuberosity

**FIGURE 2:**
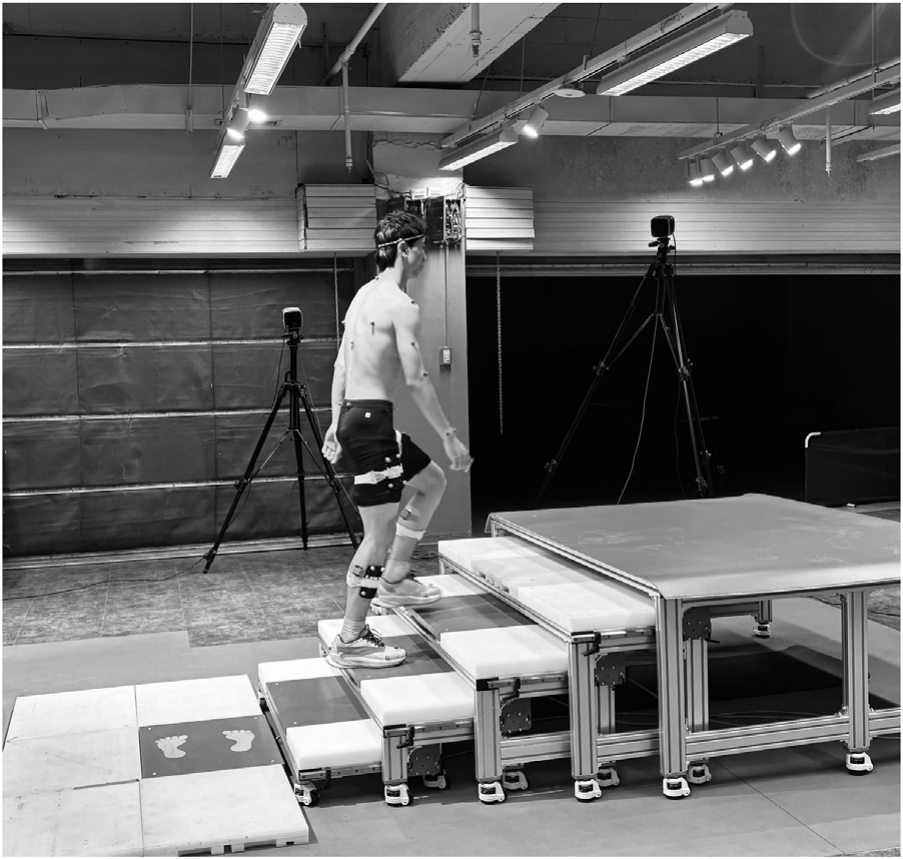
Layout of the experimental site.

The staircase comprised five steps with a 30 ± 0.05 cm tread depth and 15 ± 0.05 cm riser height. A standard stair platform customized according to the Chinese Code for Fire Protection Design of Buildings (GB 50352-2005), consisting of a five-step staircase. Four force platforms were embedded: one on the ground preceding the staircase, and three integrated into the first, second, and third steps (surfaces flush with treads). Participants performed stair ascent/descent at self-selected speeds under two loading modalities: HCC and SLC (15 kg total mass) (Simpson et al., 2012) Each movement type (ascent/descent) and loading condition (hand-carry carriage/shoulder-load carriage, HCC/SLC) combination underwent three valid trials, defined as continuous motion without pauses or marker loss. The experimental setup is depicted in Figure 2.

### 2.3 Gait cycle partitioning

**FIGURE 3:**
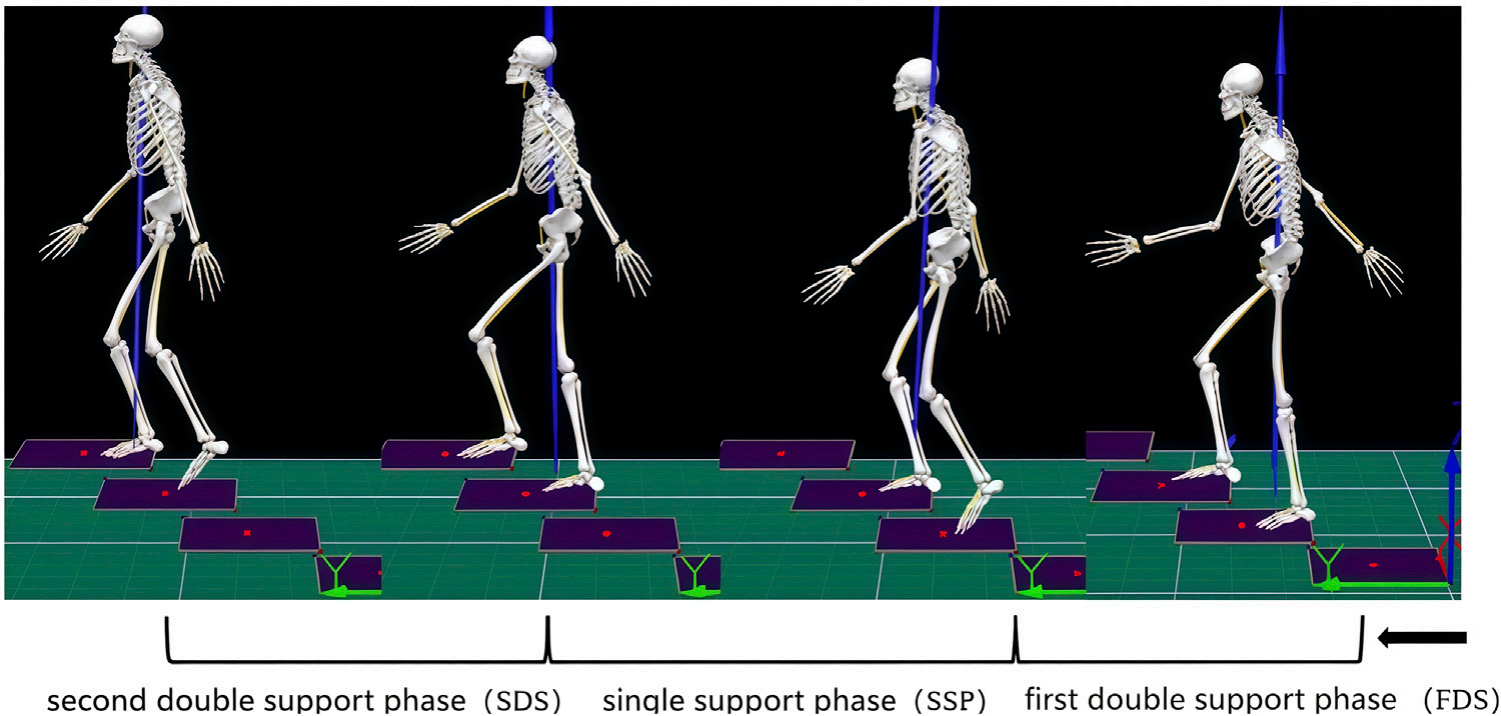
Partitioning of the gait cycle.

The gait cycle of the dominant leg’s support phase was analyzed in this study (Figure 3). A standardized kicking protocol was implemented to determine limb dominance. Participants performed three consecutive kicks toward a target placed at a 5-m distance. The dominant limb was defined as the leg used in two or more trials. The support phase was partitioned into three distinct subphases: the first double support phase (FDS), single support phase (SSP), and second double support phase (SDS). Specifically: FDS is defined as the interval starting when the right foot contacts the step and ending when the left foot completely leaves the step. SSP is defined as the period beginning when the left foot leaves the step and ending when it re-contacts the subsequent step. SDS is defined as the duration from the contact of the left foot with the step until the right foot fully leaves the step.

### 2.4 Indicator selection and data processing

Kinematic and kinetic data of the knee joint were exported *via* Visual3D and subsequently normalized to 101 data points for each gait sub-phase (first double support phase; single support phase; second double support phase) using cubic B-spline basis curves, resulting in a total of 303 data points per gait cycle. This standardization protocol minimized critical point loss while mitigating the reduction of potential inter-cluster variations, prior to SPM analysis (Warmenhoven et al., 2018). Kinematic and kinetic data were processed using Visual3D software, with all data subsequently exported to Microsoft Excel for post-processing. The inverse dynamics approach was applied to calculate net joint moments at the knee, utilizing Leva-adjusted Seluyanov’s anthropometric inertial parameters (De Leva, 1996). PFJS, COP, and CCI during the first double support phase, single support phase, and second double support phase were selected as outcome measures.

Patellofemoral mechanics-related parameters were calculated using computational models described by Bressel (Bressel, 2001) and Vannatta (Vannatta and Kernozek, 2015). The specific formulas used in these models were as follows: (1) Calculation of Quadriceps Force (QF).

$$LA=\left\{\begin{array}{l} 0.036\theta +3.0\left(0\leq \theta <30\right)\\ -0.043\theta +5.4\left(30\leq \theta <60\right)\\ -0.027\theta +4.3\left(60\leq \theta <90\right)\\ 2.0\left(90\leq \theta \right) \end{array}\right.$$ (1)

QF was calculated as the ratio of the knee extension moment to the effective moment arm of the quadriceps. In Equation 1, LA (effective moment arm of the quadriceps) is expressed as a piecewise function of the sagittal plane knee angle (KA, denoted as θ ).

$$M_{\text{EXT}=}M_{\text{NET}}$$ (2)

$$\text{QF}\left(\theta_{i}\right)=M_{\text{EXT}}\left(\theta_{i}\right)/\left[\text{LA}\left(\theta_{i}\right)*0.01\right]$$ (3)

In Equation 2: $M_{EXT}$ (N·m) represents the sagittal plane knee extension moment. ${\;M}_{\text{NET}}$ (N·m) denotes the net knee moment in the sagittal plane. In Equation 3: ${\;\theta }_{i}\;\left({}^{\circ}\right)$ corresponds to the knee flexion-extension angle at the *i*th frame.

(2) Calculation of Patellofemoral Joint Force (PFJF)

$$\beta =30.46+0.53\left(\theta \right)$$ (4)

$$\text{PFJF}=2QF_{\sin}\left(\beta /2\right)$$ (5)

The patellofemoral joint force (PFJF) was calculated using Equations 4, 5, where β (°) denotes the angle between the line of action of the quadriceps force and the patellar tendon force (Bressel, 2001).

(3) Calculation of Patellofemoral Joint Stress (PFJS)

$$\text{PFCA}\left(\theta_{i}\right)=0.078\times \theta_{i}^{2}+0.6763\times \theta_{i}+151.75$$ (6)

$$\text{PFJS}\left(\theta_{i}\right)=\text{PFJF}\left(\theta_{i}\right)/\text{PFCA}\left(\theta_{i}\right)$$ (7)

The patellofemoral contact area (PFCA) was modeled as a function of the sagittal plane knee angle θ and calculated using Equation 6. PFJS was then determined as the ratio of PFJF to PFCA (Equation 7) (Bressel, 2001).

Co-cativation indices were quantified based on electromyography activity ratios between antagonist and agonist muscle pairs. During stair ascent, the following muscle pairs were analyzed: (1) In the lower leg during the FDS and SSP, the TA acted as the antagonist opposing the agonist pair of MG and LG; (2) in the thigh during these phases, the BF served as the antagonist against the agonist group comprising the VM, RF, and VL. During the SDS of ascent, however, the agonist-antagonist roles in the lower leg were reversed, with the MG and LG becoming antagonists and the TA acting as the agonist. During stair descent, the antagonist-agonist roles of all muscle pairs were systematically reversed compared to their roles during ascent phases.

$$\text{Co}-\text{cativation}=\frac{{RMS}_{antagonists}}{{RMS}_{agonists}}$$

### 2.5 Statistical analysis

For continuous variables, SPM two-sample t-tests were employed to analyze curve data, with the significance level set at *P* < 0.05, to compare differences in PFJS between HCC and SLC modes across distinct gait phases. Between-group differences in COP and CCI were evaluated via independent t-tests in SPSS 27.0, with significance set at *P* < 0.05. Data organization, statistical analysis, and graphing were performed using Microsoft Excel 2021 and Origin Pro 2024. SPM analysis was conducted using the open-source spm1d package (www.spm1d.org) within the OriginPro 2024 environment.

## 3 Results

### 3.1 Biomechanical characteristics of PFJS

**FIGURE 4:**
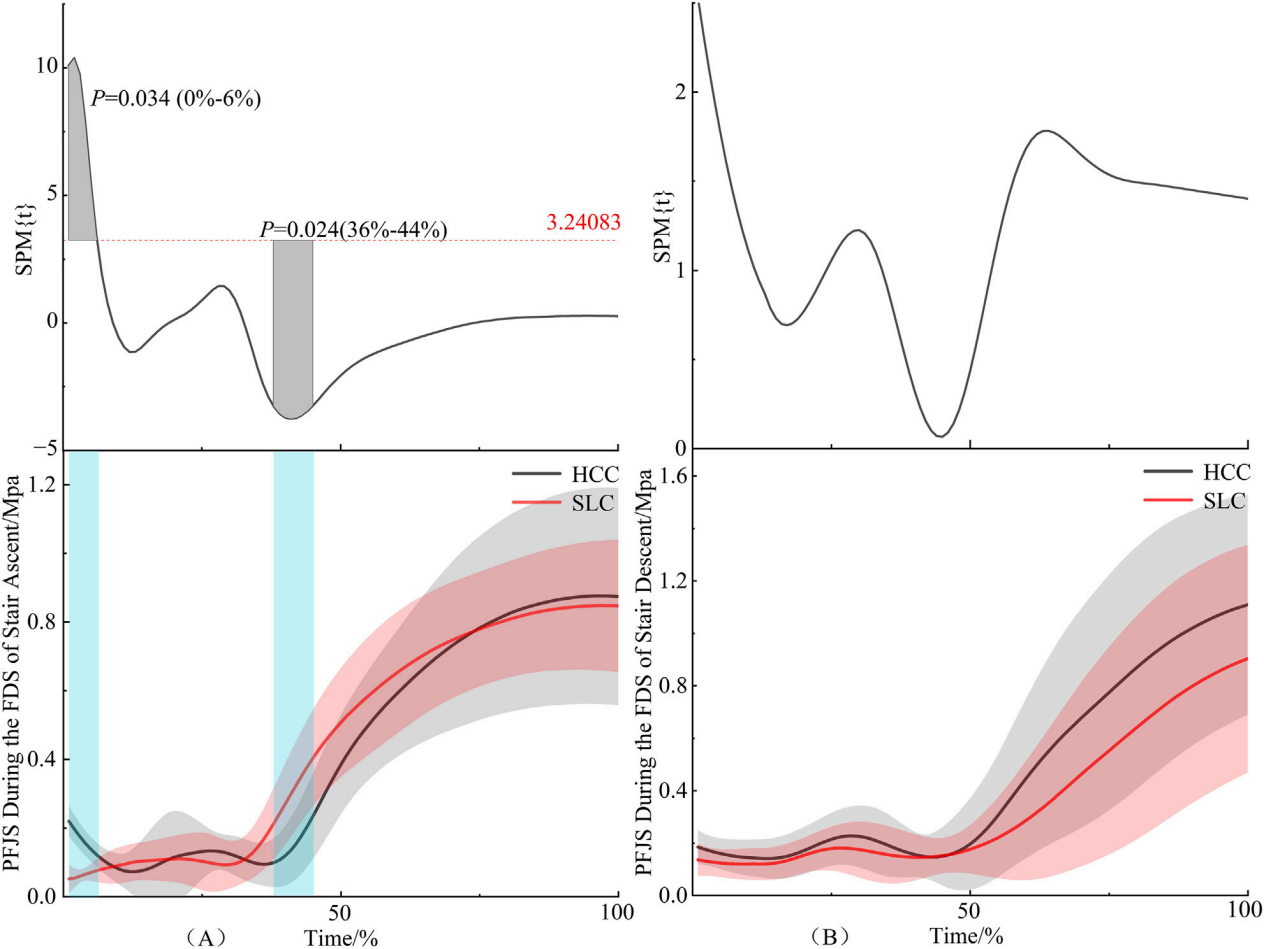
PFJS during the first double support phase. Note: Blue shaded areas indicate differences. **(A)** denotes stair ascent, **(B)** denotes stair descent. The same applies below.

As illustrated in Figure 4A, the PFJS under HCC exhibited a significant elevation compared to SLC during the 0%–6% interval of the first double support phase in stair ascent (*P* = 0.034). Conversely, PFJS values under HCC were significantly reduced relative to SLC within the 36%–44% interval (*P* = 0.024). Figure 4B shows no significant difference during the first double support phase of stair descent.

**FIGURE 5:**
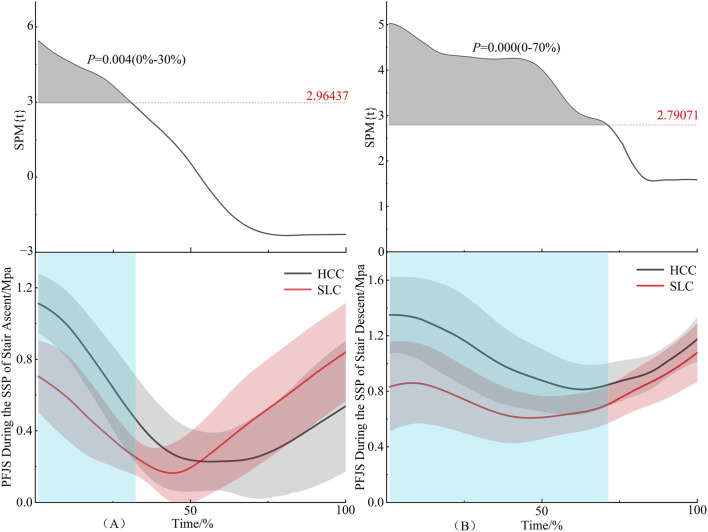
PFJS during the single support phase. **(A)** denotes stair ascent, **(B)** denotes stair descent.

As illustrated in Figure 5A, HCC induced significantly higher PFJS than SLC during the 0%–30% interval of the single support phase in stair ascent (*P* = 0.004). Correspondingly, Figure 5B reveals that PFJS under HCC remained elevated relative to SLC across the 0%–70% interval of the single support phase during stair descent (*P* = 0.000).

**FIGURE 6:**
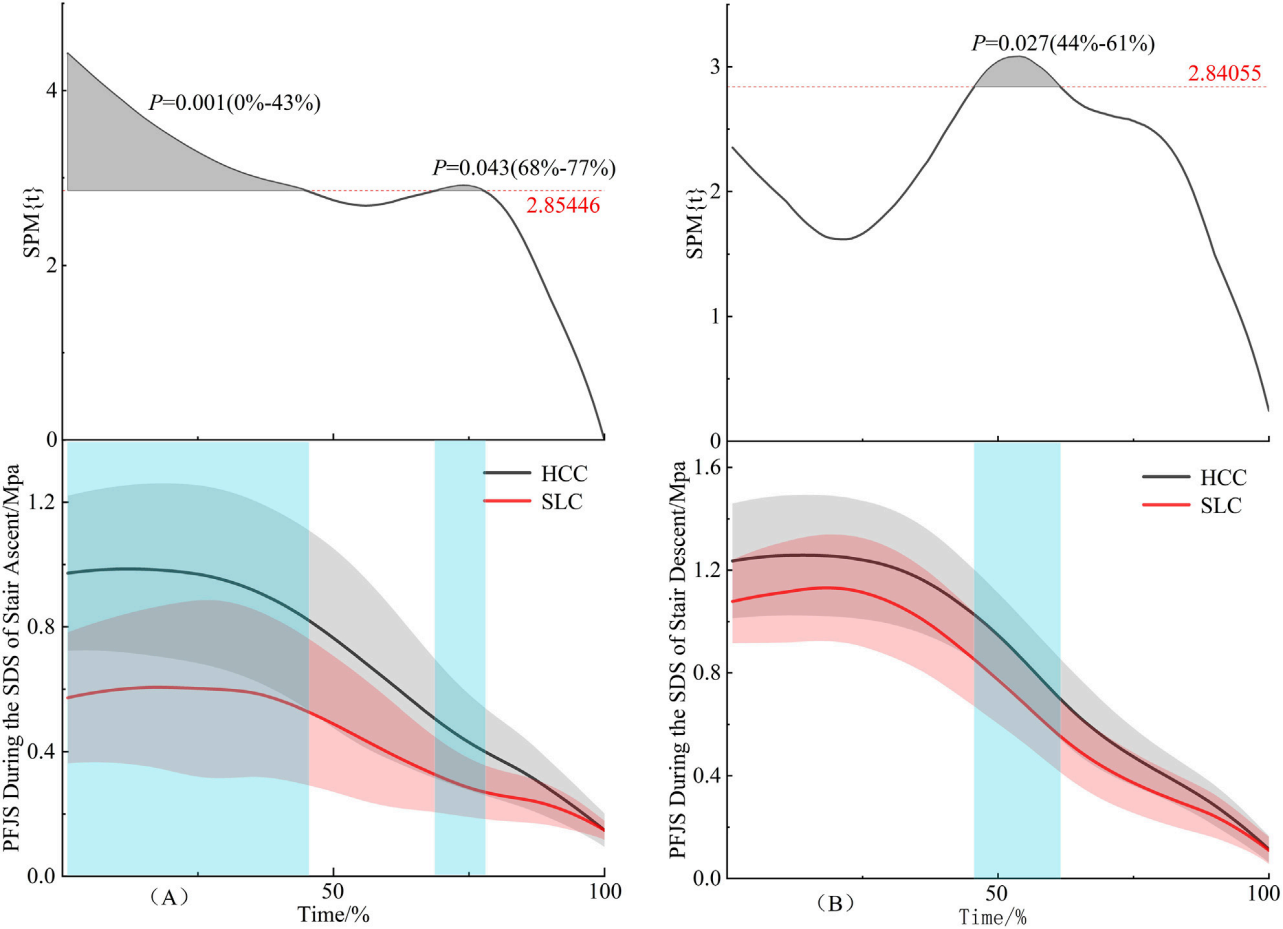
PFJS during the second double support phase. **(A)** denotes stair ascent, **(B)** denotes stair descent.

As illustrated in Figure 6A, the HCC induced significantly higher PFJS than the SLC during 0%–43% interval (*P* = 0.001) and 68%–77% interval (*P* = 0.043) of the second double support phase in stair ascent. Figure 6B further demonstrates that HCC generated elevated PFJS compared to SLC within the 44%–61% interval of the second double support phase during stair descent (*P* = 0.027).

**FIGURE 7:**
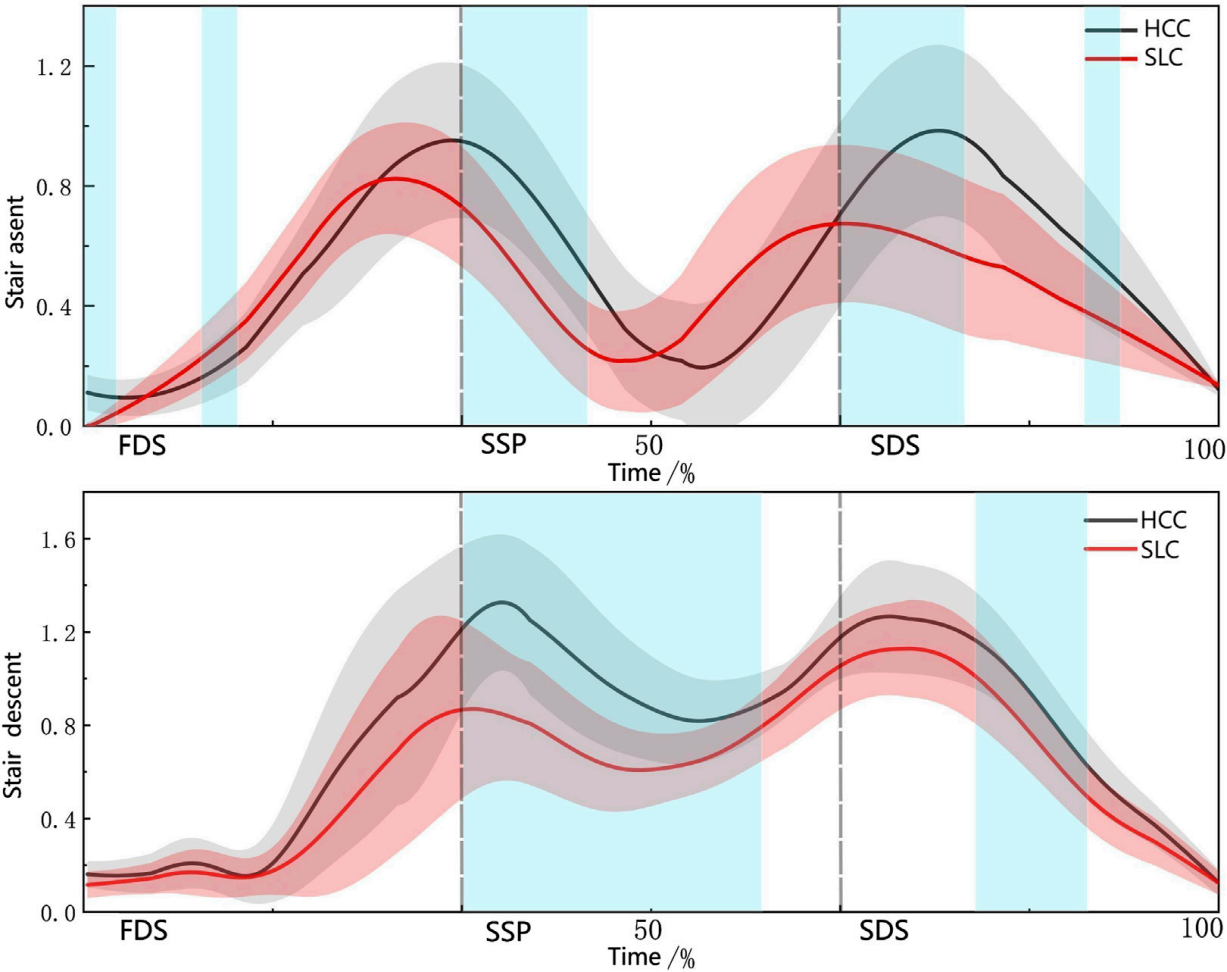
PFJS trajectories during the support phase.

As illustrated in Figure 7, both load carriage modalities—HCC and SLC—exhibited biphasic fluctuations in PFJS during stair negotiation, characterized by two distinct peaks and troughs. Notably, transient PFJS elevations were observed during gait phase transitions (e.g., double-to-single support shifts) across both tasks.

### 3.2 Duration of statistically significant differences across gait phases

**FIGURE 8:**
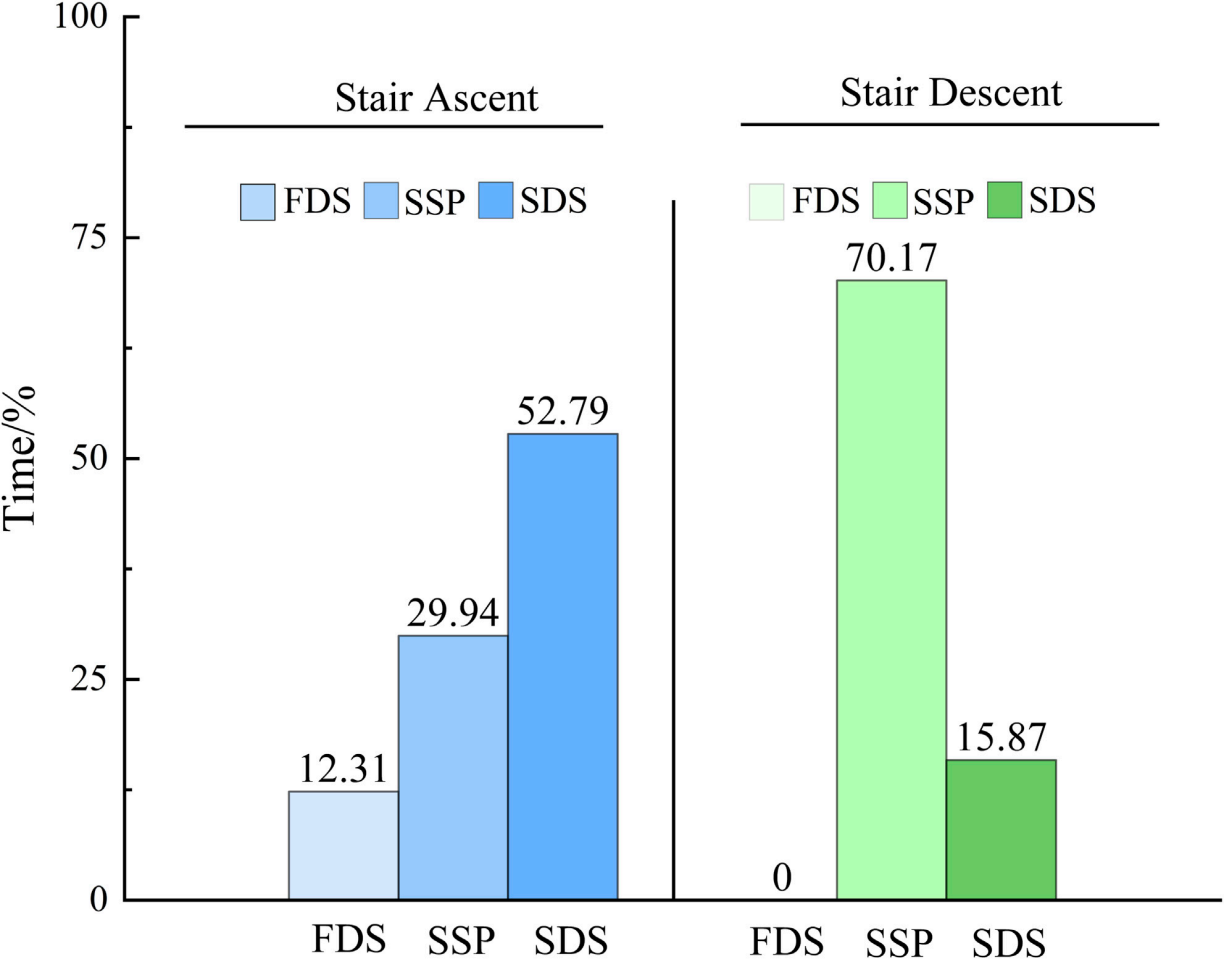
Comparison of duration.

As illustrated in Figure 8, inter-mode PFJS divergence durations exhibited task and phase dependent variability: the longest duration of PFJS differences was observed during the single support phase of stair descent (70.17% of phase duration), followed sequentially by the second double support phase in stair ascent (52.79%), the single support phase in stair ascent (29.94%), the second double support phase in stair descent (15.87%), and the first double support phase in stair ascent (12.31%).

### 3.3 COP displacement characteristics

As shown in Table 2, the COP amplitude under HCC significantly exceeded that under SLC during the first double support phase of stair ascent (SA) (*P* = 0.022). During stair descent (SD), HCC generated larger anteroposterior (AP) and mediolateral (ML) COP displacements in the first double support phase and higher ML COP variability in the single support phase compared to SLC (*P* = 0.031; *P* = 0.004; *P* = 0.020).

**TABLE 2:** Center of pressure.

Cop		FDS		SSP		SDS	
		HCC	SLC	HCC	SLC	HCC	SLC
SA	AP	0.058 ± 0.019*	0.041 ± 0.022	0.018 ± 0.008	0.015 ± 0.009	0.019 ± 0.009	0.017 ± 0.014
	ML	0.074 ± 0.036	0.068 ± 0.033	0.059 ± 0.027	0.064 ± 0.024	0.067 ± 0.025	0.064 ± 0.029
SD	AP	0.073 ± 0.019*	0.059 ± 0.016	0.017 ± 0.009	0.016 ± 0.007	0.016 ± 0.009	0.016 ± 0.010
	ML	0.125 ± 0.022*	0.102 ± 0.021	0.065 ± 0.020*	0.049 ± 0.017	0.060 ± 0.029	0.068 ± 0.017

Note: * denotes *P* < 0.05.

### 3.4 Differences in Co-cativation indices

**FIGURE 9:**
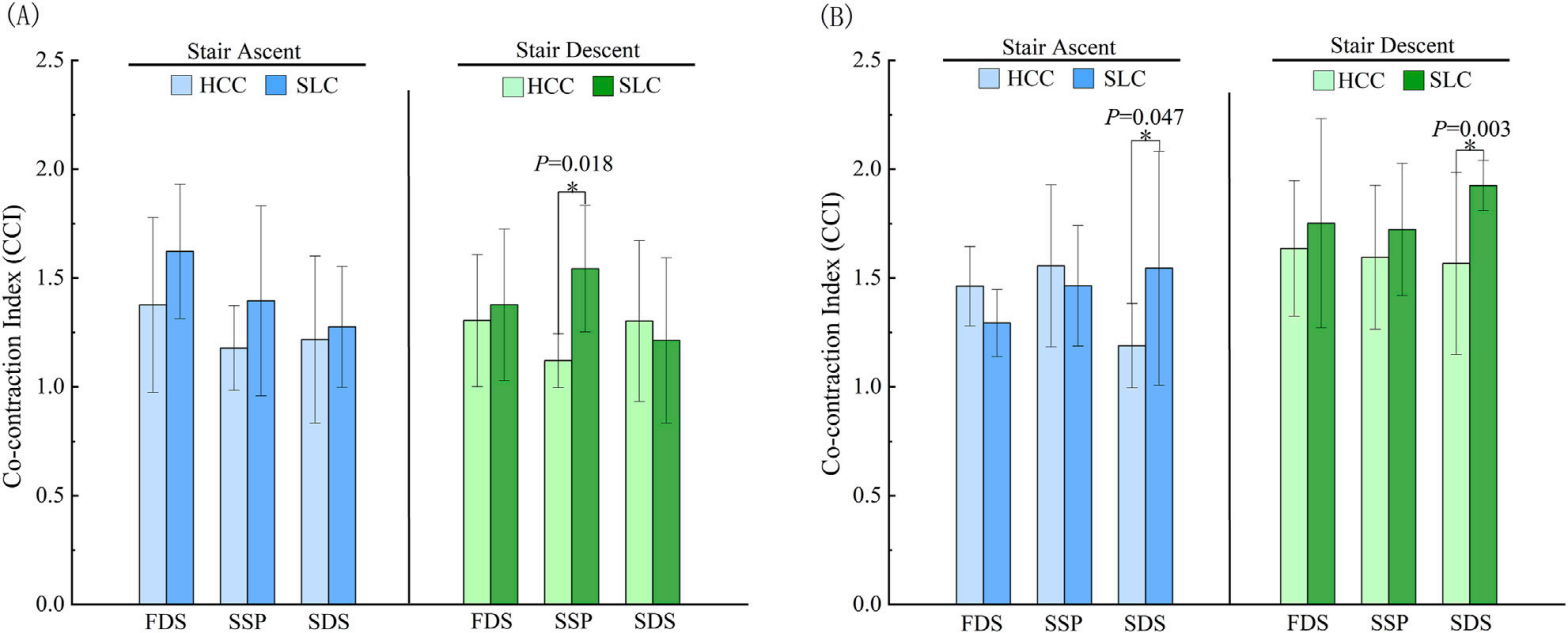
Co-cativation indices. Note: **(A)** denotes calf muscles, **(B)** denotes thigh muscles.

As shown in Figure 9A, the lower leg co-cativation indices (CCI) under HCC was significantly lower than that under SLC during the single support phase of stair descent (*P* = 0.018). Similarly, Figure 9B demonstrates that HCC exhibited markedly reduced thigh CCI compared to SLC in the second double support phase during stair negotiation (*P* = 0.047; *P* = 0.003).

## 4 Discussion

Excessive Patellofemoral joint stress (PFJS) is a key factor in triggering patellofemoral pain (Adebayo et al., 2019; Kaila-Kangas L. et al., 2011). Biomechanical studies indicate that increased PFJS primarily results from elevated contact forces or reduced contact area. The study revealed that PFJS was significantly higher during the mid-phase of the first double support phase under shoulder-load carriage (SLC). Further analysis revealed that the transient PFJS elevation during the first double support phase under SLC might be associated with postural compensatory mechanisms. Specifically, alterations in the body center of mass (COM) position altered lower limb kinetic chain alignment, inducing reorientation of patellofemoral force vectors along with amplified vertical joint reaction forces, thereby elevating patellofemoral contact pressure (Na et al., 2020).

The phase analysis of this study revealed that although SLC generates higher instantaneous stresses during the first double support phase, their impact is confined to a single phase. When analyzed over the entire gait cycle, HCC pose greater biomechanical risks to the patellofemoral joint. During both the single support phase and the second double support phase of stair gait, the patellofemoral joint experiences significantly greater pressure under hand-carrying conditions compared to shoulder-loading. This suggests that hand-carry carriage imposes higher impact loading on the patellofemoral joint, presenting elevated cumulative injury risks relative to shoulder-loading methods. These conclusions are substantiated not only by differences in stress distribution patterns, but also by the significant influence of loading modalities on body stability and neuromuscular coordination mechanisms.

The study found that carrying loads by HCC during stair negotiation leads to a significant increase in center of pressure (COP) displacement distance. This parameter change can serve as an objective indicator of reduced postural control ability (Vieira et al., 2021) especially during the single support phase and the second double support phase, where COP deviations in anteroposterior and mediolateral directions become more pronounced. Further analysis reveals that when carrying loads by hand, the load positioned far from the body’s center of gravity forces the trunk to compensate by leaning forward, sideways, or backward to maintain balance, disrupting the original dynamic stability. When lower limb postural stability is poor, this may affect the mechanical alignment of the knee joint, leading to uneven stress distribution and increased patellofemoral joint stress (FanTing and Zhang, 2023). In comparison, the greater stability observed during SLC is likely attributed to the load is closer to the midline of the body’s trunk, with the gravity line closer to the supporting foot, thereby reducing lateral COP displacement (Vieira et al., 2021).

The changes in muscle co-activation characteristics provide a deeper explanation for this phenomenon. When carrying loads by hand during the single-support phase of stair descent, the co-activation index (CCI) of the lower leg and the thigh CCI during the double-support phase were both significantly lower than when using shoulder-load carriage, but the PFJS was higher. Lower co-activation indices indicate weakened muscular synergistic capacity, particularly insufficient hamstring activation failing to effectively counterbalance the quadriceps' tensile direction. This results in abnormal patellar tracking (e.g., lateral displacement) and reduced contact area (Salsich et al., 2003), which subsequently leads to localized stress concentration (Watson et al., 2017). Meanwhile, the study observed that during HCC, the weight suspended vertically at the body side shifts the load’s center of gravity forward. This prolongs the moment arm from the knee flexion axis to the load, and the elongated lever arm increasing quadriceps contraction force required for equilibrium maintenance, thereby elevating pressure between the patella and femur (Brechter and Powers, 2002). In contrast, during SLC, the center of gravity is closer to the torso’s midline, shortening external moment arms. This mechanical advantage may mitigate the adverse effects of higher co-activation indices on joint stress.

More importantly, the single support phase during stair descent represents the period with the poorest dynamic stability and the longest duration of discrepancy, corroborating previous research findings that load carrying significantly impacts gait, with increased loading leads to corresponding reductions in gait stability (Demura et al., 2010). HCC further compromises the body’s balance maintenance capacity. Under asymmetric loading conditions, elevated lateral shear forces exacerbate balance control deficits (Seriani et al., 2021), compounded by amplified COP displacement and insufficient muscle co-activation, forming a “reduced stability-increased stress” vicious cycle: gravity shift exacerbates patellofemoral joint malalignment, while low co-activation indices weaken mediolateral patellar stability, ultimately resulting in significantly elevated stress. Consistent with the conclusion of Bosse et al. (2012) that stability reaches its minimum during support phase transitions, our findings also confirm increased PFJS during these transitions, indicating that loading methods and weight may amplify instability, further impairing patellofemoral joint stability and elevating stress. This mechanism strongly aligns with Crossley K M. et al. (2016) progressive injury model of PFPS, where repetitive mechanical stress induces progressive accumulation of cartilage matrix microdamage, ultimately triggering pain syndromes.

On the other hand, HCC as a unilateral loading mode is closely associated with kinetic chain compensation. Paillard (2012) and Baggaley et al. (2020) proposed that external loads displace the body’s center of gravity, disrupting inherent dynamic equilibrium and negatively affecting both sensory inputs for postural control and motor outputs, thereby inducing compensatory postural adaptations. Such multi-joint linkage imbalance weakens muscular synergistic capacity, accelerates fatigue in lower leg muscle groups, and forces compensatory knee joint mechanics, ultimately increasing PFJS (Baggaley et al., 2020). Integrated with COP and muscle co-activation findings, hand-carry carriage not only directly augments joint torque through lever arm effects but also indirectly amplifies stress by impairing stability and neuromuscular coordination, thereby establishing injury risks through multiple synergistic pathways. In addition, previous studies have indicated that asymmetric muscle activation patterns during trunk movement can also lead to low back pain (Ng et al., 2002). When asymmetric loading causes the total center of mass of the body and load to shift, it recruits more lower limb muscles to maintain balance, resulting in increased contraction forces in the lower limb muscles—particularly the quadriceps. External loads amplify joint torque through lever arms, pulling the patella and increasing its contact force with the femur, thereby elevating patellofemoral joint stress. Moreover, trunk rotation and lateral flexion activate different trunk muscle groups, triggering compensatory responses in specific antagonistic or contractile muscle groups, which heighten the likelihood of low back pain (Gonehe and Feng, 2020).

The findings prompt recommendations to optimize loading methods (proximal loading or bilateral loading) or enhance quadriceps muscle strength through targeted exercises (particularly the vastus medialis) to improve patellar stability, thereby reducing injury risks in both the lower back and patellofemoral joint. In summary, hand-carrying imposes greater patellofemoral joint stress due to the synergistic effects of multiple mechanisms: increased COP displacement, insufficient muscle co-activation, and lever arm elongation.

## 5 Conclusion

Distinct load carriage modes exert significant effects on the biomechanical behavior of the patellofemoral joint. Compared to SLC, HCC significantly increases PFJS during both the single support phase and the second double support phase of stair negotiation. This biomechanical risk escalation is mechanistically linked to two synergistic factors: (1) amplified COP displacement in anteroposterior and mediolateral directions, and (2) reduced lower limb CCI—both collectively exacerbating dynamic joint stability deterioration under loaded stair negotiation tasks. While SLC generates transient stress peaks during the first double support phase, it maintains global kinetic chain equilibrium through external moment arm reduction. Conversely, HCC—characterized by asymmetric loading—prolongs the knee joint moment arm via COM anterior displacement, thereby compelling quadriceps overactivation and inducing abnormal patellar tracking kinematics. This biomechanical cascade amplifies PFJS through lateralized force vector realignment and sustained cartilage overload, posing risks of progressive degenerative injury in repetitive stair negotiation tasks. We recommend implementing proximal symmetrical load carriage strategies (e.g., bilateral shoulder loading) combined with targeted quadriceps strengthening exercises, particularly focusing on the vastus medialis oblique, to enhance patellar dynamic stability and mitigate risks of patellofemoral joint pain and low back pain during occupational/recreational stair negotiation tasks.

## 6 Limitations

Furthermore, this study has several limitations. First, the investigation was restricted to male participants and adopted a single load condition (15 kg) during standardized stair-climbing tasks, failing to simulate real-world complexities such as sudden stops or directional changes. Consequently, the conclusions are strictly valid for stair negotiation tasks under 15 kg hand-carried (HCC) and shoulder-loaded (SLC) conditions in young males. While results might be cautiously extrapolated to comparable load ranges (10–20 kg), the effects of different load magnitudes, gender disparities, and their applicability to broader populations (e.g., females, older adults) or extreme loads (more than 25 kg) remain unverified and require systematic exploration through further validation studies.

## Data Availability

The original contributions presented in the study are included in the article/supplementary material, further inquiries can be directed to the corresponding authors.

## Abbreviations

FDS, The first double support phase; SSP, the single support phase; SDS, the second double support phase; SPM, Statistical Parametric Mapping; SLC, shoulder-load carriage; HCC, hand-carry carriage; CCI, co-cativation indices; PFJS, patellofemoral joint stress; COM, center of mass; COP, the center of pressure.
